# Effects of bowel preparation on the human gut microbiome and metabolome

**DOI:** 10.1038/s41598-019-40182-9

**Published:** 2019-03-11

**Authors:** Naoyoshi Nagata, Mari Tohya, Shinji Fukuda, Wataru Suda, Suguru Nishijima, Fumihiko Takeuchi, Mitsuru Ohsugi, Tetsuro Tsujimoto, Tomoka Nakamura, Akira Shimomura, Naohiro Yanagisawa, Yuya Hisada, Kazuhiro Watanabe, Koh Imbe, Junichi Akiyama, Masashi Mizokami, Tohru Miyoshi-Akiyama, Naomi Uemura, Masahira Hattori

**Affiliations:** 1 Department of Gastroenterology and Hepatology, National Center for Global Health and Medicine, Tokyo, Japan; 2 Pathogenic Microbe Laboratory, Research Institute, National Center for Global Health and Medicine, Tokyo, Japan; 3 Department of Microbiology, Juntendo University School of Medicine, Tokyo, Japan; 4 Institute for Advanced Biosciences, Keio University, Tokyo, Japan; 5 Intestinal Microbiota Project, Kanagawa Institute of Industrial Science and Technology, Ebina, Japan; 6 Transborder Medical Research Center, University of Tsukuba, Tsukuba, Japan; 7 PRESTO, Japan Science and Technology Agency, Kawaguchi, Japan; 8 Department of Gene Diagnostics and Therapeutics, National Center for Global Health and Medicine, Tokyo, Japan; 9 Computational Bio-Big Data Open Innovation Lab., National Institute of Advanced Science and Technology, Tokyo, Japan; 10 Graduate School of Advanced Science and Engineering, Waseda University, Tokyo, Japan; 11 RIKEN Center for Integrative Medical Sciences, Wako, Japan; 12 Department of Diabetes, Endocrinology, and Metabolism, Center Hospital, National Center for Global Health and Medicine, Tokyo, Japan; 13 Department of Gastroenterology and Hepatology, National Center for Global Health and Medicine, Kohnodai Hospital, Tokyo, Japan

## Abstract

Large bowel preparation may cause a substantial change in the gut microbiota and metabolites. Here, we included a bowel prep group and a no-procedure control group and evaluated the effects of bowel prep on the stability of the gut microbiome and metabolome as well as on recovery. Gut microbiota and metabolome compositions were analyzed by 16S rRNA sequencing and capillary electrophoresis time-of-flight mass spectrometry, respectively. Analysis of coefficients at the genus and species level and weighted UniFrac distance showed that, compared with controls, microbiota composition was significantly reduced immediately after the prep but not at 14 days after it. For the gut metabolome profiles, correlation coefficients between before and immediately after the prep were significantly lower than those between before and 14 days after prep and were not significantly different compared with those for between-subject differences. Thirty-two metabolites were significantly changed before and immediately after the prep, but these metabolites recovered within 14 days. In conclusion, bowel preparation has a profound effect on the gut microbiome and metabolome, but the overall composition recovers to baseline within 14 days. To properly conduct studies of the human gut microbiome and metabolome, fecal sampling should be avoided immediately after bowel prep.

## Introduction

Adequate bowel cleansing is essential for diagnosing gastrointestinal disease accurately on colonoscopy and for ensuring the safety of therapeutic endoscopy^1^. Several studies have shown the efficacy and safety of bowel preparation^1^, but it is also likely to be a major disrupter of the colonic ecosystem. As high-volume lavage solution passes rapidly through the bowel tract, increasing the frequency and force of colonic peristalsis and changing stool consistency^1–3^, it may wash out the luminal content and cause a substantial change in the gut microbiota and metabolites.

No consensus has been reached on the duration of effect of bowel prep on gut microbiota. Four studies have shown significant changes in fecal microbial composition or diversity before and after high-volume lavage^3–6^. One of these studies demonstrated a long-lasting (1-month) effect on gut microbiota composition and homeostasis, and especially a reduction in *Lactobacillaceae* abundance^5^. Bowel prep also affects the composition and diversity of mucosal-adherent microbiota, as determined from colonoscopic biopsy samples^4,6^. In contrast, the other three studies suggested that high-volume lavage does not alter microbial diversity over the long term^7–9^, although two of the three did show a significant change in gut microbiota immediately after bowel prep^7,9^. These conflicting results are probably due to the small number of subjects, the inclusion of healthy and diseased subjects, the lack of a non-procedure control group, and a lack of analytical depth in these studies.

Recently, there has been increasing recognition that microbiota-generated metabolites are an essential part of human physiology, and that the microbiome-metabolome interaction is closely involved in human health and disease^10^. However, no studies so far have evaluated the effects of bowel prep on the gut metabolome.

In this study, we determined changes in the gut microbiota and metabolites associated with bowel prep using 16S ribosomal RNA (rRNA) sequencing and capillary electrophoresis time-of-flight mass spectrometry (CE-TOF-MS). Specifically, we sought to determine the immediate effects of bowel prep on the stability of the gut microbiome and metabolome as well as the impact on microbial and metabolic recovery.

## Results

**Figure 1:**
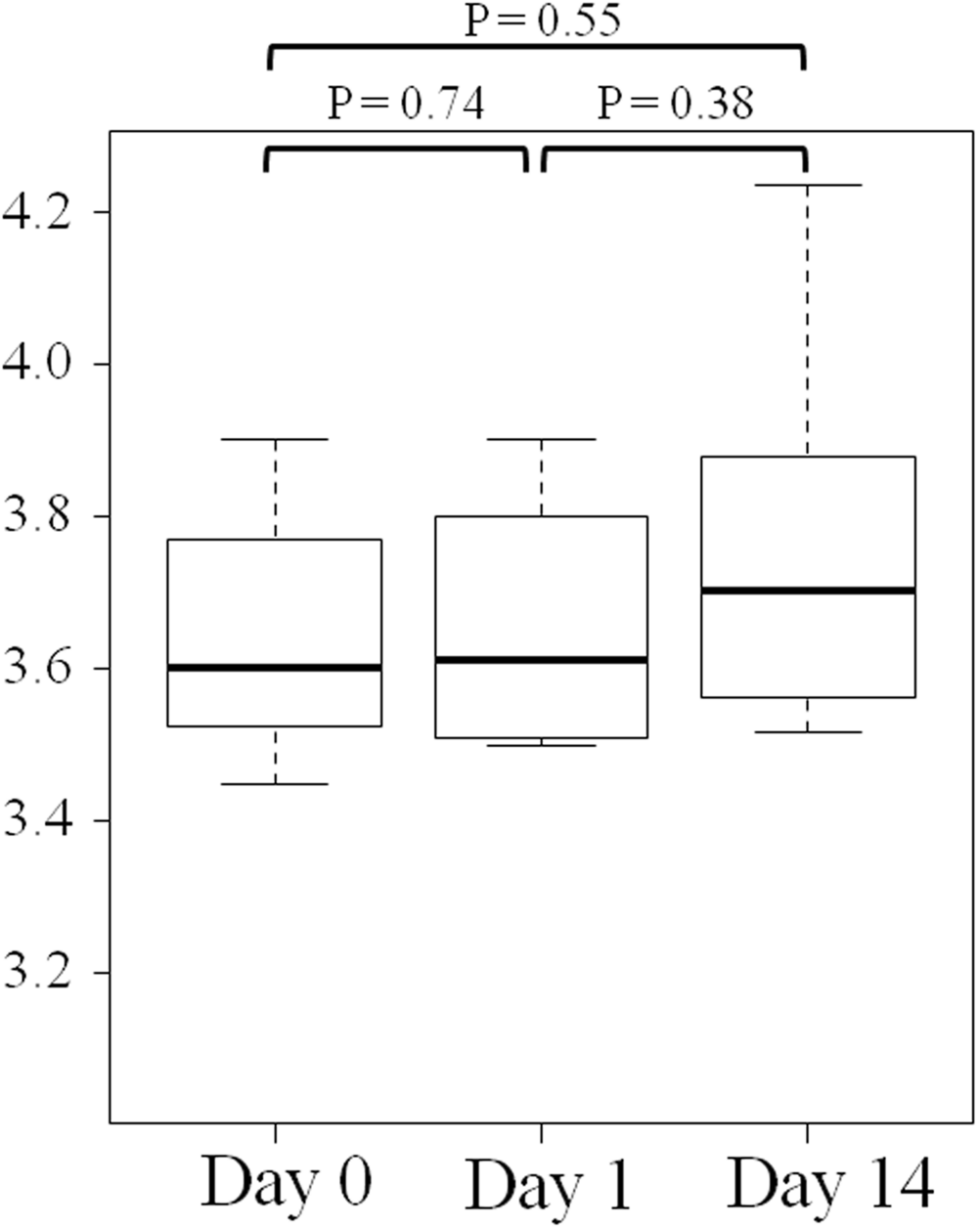
Gut microbiota α-diversity as measured by the Shannon index before, during, and after bowel preparation. Note: Boxes represent the interquartile range (IQR) and lines inside show the median. Whiskers denote the lowest and highest values within 1.5 times the IQR.

**Figure 2:**
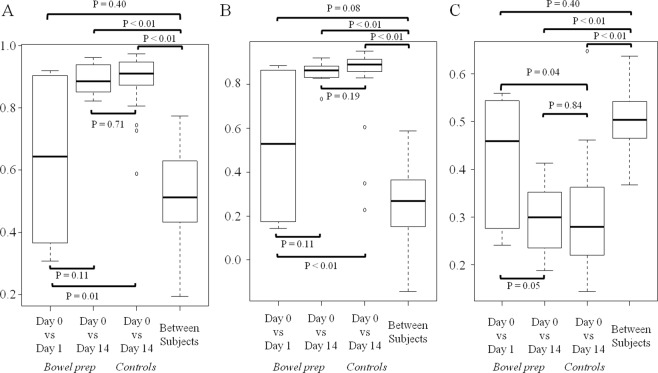
Gut microbial composition across different sampling times in the bowel prep group vs control group. (**A**) Spearman’s correlation coefficients of gut microbial composition at the genus level. (**B**) Spearman’s correlation coefficients of gut microbial composition at the species level. (**C**) Weighted UniFrac distances of gut microbial composition. Note: Boxes represent the interquartile range (IQR) and lines inside show the median. Whiskers denote the lowest and highest values within 1.5 times the IQR.

A total of 70 fecal samples (24 in the bowel prep group and 46 in the control group) were analyzed. α-diversity of gut microbiota was not significantly different between Day 0, Day 1, and Day 14 samples in the bowel prep group (Fig. 1). At the genus level, Spearman’s correlation coefficients between regular feces (Day 0) and first feces immediately after bowel prep (Day 1) were significantly lower than that of controls (Fig. 2A), whereas coefficients between Day 0 and Day 14 samples did not differ significantly from the controls (Fig. 2A). Spearman’s correlation coefficients between Day 0 and Day 1 samples were not significantly different compared with those for between-subject differences (Fig. 2A). Similar results were observed at the species level (Fig. 2B). Likewise, weighted UniFrac distances between Day 0 and Day 1 samples were significantly larger than those of controls (Fig. 2C), whereas distances between Day 0 and Day 14 samples were not significantly different from the controls (Fig. 2C). Weighted UniFrac distances between Day 0 and Day 1 samples were not significantly different compared with those for between-subject differences (Fig. 2C).

**Figure 3:**
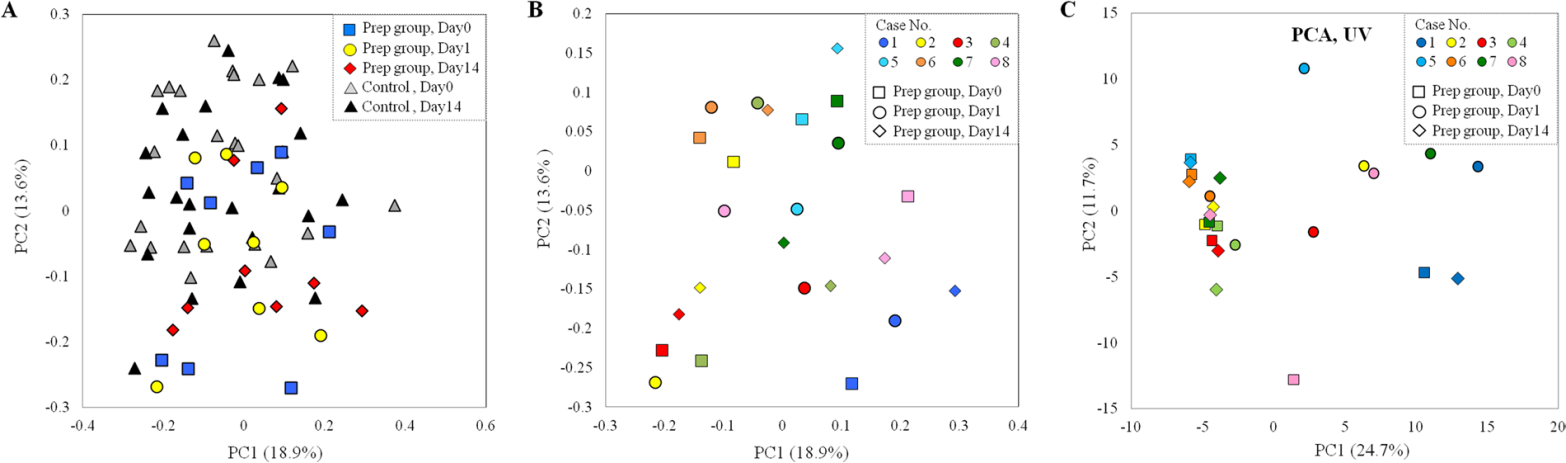
Principal coordinate analysis (PCoA) plot in microbiome and metabolome. (**A**) PCoA plot of weighted UniFrac distances for samples between bowel prep group and controls in microbioal analysis. (**B**) PCoA plot of weighted UniFrac distances for samples in bowel prep group in microbial analysis. (**C**) PCoA plot, unit variance scaling in bowel prep group in metabolome analysis.

Principle coordinate analysis (PCoA) of the weighted UniFrac distances of the samples revealed that they were not divided into separate clusters between the two groups (Fig. 3A), suggesting a small impact on the microbiome analysis. In the bowel prep group, the PCoA plot clustered all individuals except for case 4, but it did not cluster all individuals for timing (Fig. 3B).

The relative abundances of four phyla did not change significantly in regular feces (Day 0), first feces immediately after bowel prep (Day 1), or feces at 14 days after bowel prep (Day 14) (Supplementary Fig. 1**)**. The predominant 38 genera with >1% relative abundance did not change significantly between Day 0 and Day 1 or between Day 1 and Day 14 **(**Supplementary Table 1**)**. Only *Streptococcus* was significantly different between Day 1 and Day 14 (p = 0.022), but this change was not significant after FDR correction (q = 0.520) **(**Supplementary Table 1**)**. A total of 127 operational taxonomic units (OTUs) were identified from the gut microbial population representing >0.1% relative abundance. Of these OTUs, three OTUs (OTU-29 *Bacteroides ovatus*, OTU-163 *Bacteroides thetaiotaomicron*, and OTU-585 *Clostridium cellulolyticum*) were significantly changed on Day 1 compared with Day 0 (Supplementary Table 2**)**. In control group, two OTUs (OTU-16 *Eubacterium ramulus* and OTU-47 *Clostridium sp*.) were significantly changed between Day 1 and Day 14 (Supplementary Table 2**)**. However, these results were not significant after FDR correction (Supplementary Table 2**)**. The number of changed OTUs was significantly larger on Day 1 than on Day 14 for ≥2-, 3-, 5-, and 10-fold changes (Supplementary Fig. 2).

**Figure 4:**
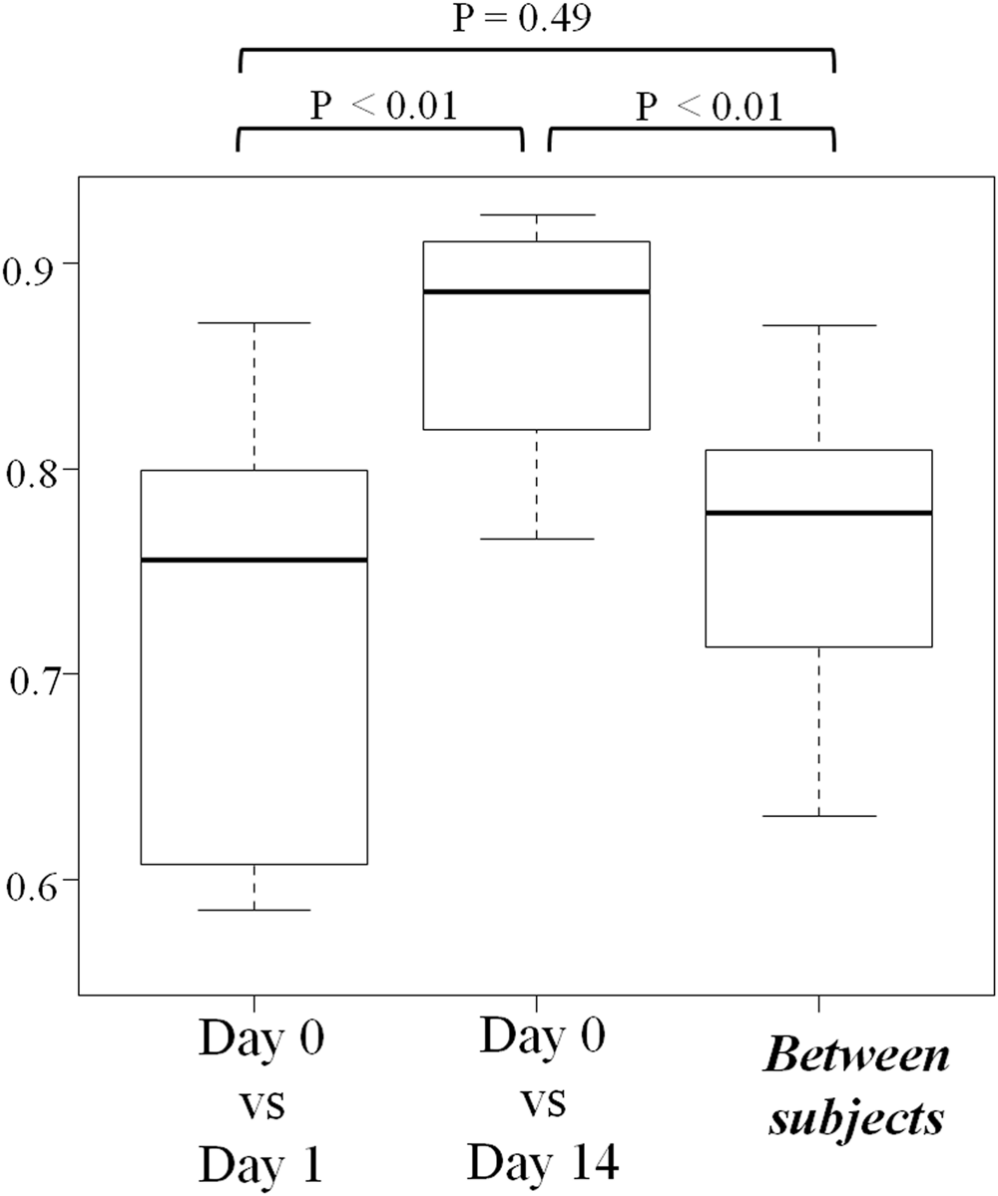
Spearman’s correlation coefficients of fecal metabolome profiles before bowel prep (Day 0), immediately after bowel prep (Day 1), and 14 days after bowel prep (Day 14). Note: Boxes represent the interquartile range (IQR) and lines inside show the median. Whiskers denote the lowest and highest values within 1.5 times the IQR.

For the gut metabolome profiles, Spearman’s correlation coefficients between Day 0 (regular feces) and Day 1 (first feces immediately after bowel prep) were significantly lower than those between Day 0 and Day 14 samples (Fig. 4). Spearman’s correlation coefficients between Day 0 and Day 1 samples were not significantly different compared with those for between-subject differences (Fig. 4). PCoA unit variance scaling revealed that the plot clustered all individuals except cases 7 and 8. In cases 1, 2, 3, 5, and 7, the metabolome profiles changed in the Day 1 samples, whereas the Day 0 and Day 14 samples were clustered in these cases (Fig. 3C). We measured 514 metabolites in fecal samples using CE-TOF-MS (Supplementary Table 3). Of these, 32 metabolites were significantly changed on Day 1 (immediately after bowel prep) compared with Day 0 (Table 1). However, they recovered within 14 days of bowel prep (Day 0 vs Day 14) (Table 1).

**Table 1:** Significant changes of metabolites before prep (Day 0), immediately after prep (Day 1), and 14 days after prep (Day14).

Name	Day0			Day1			Day14			Day0 vs Day1		Day0 vs Day14		Day1 vs Day14	
	median	min	max	median	min	max	median	min	max	p-value	q-value	p-value	q-value	p-value	q-value
Ala	2150.9	790.0	31602.1	18956.5	3482.8	37939.2	1948.3	490.9	39944.5	0.0078	0.1484	0.8438	1.0000	0.0547	0.2528
Azelate	94.7	43.3	206.0	50.5	17.2	86.4	82.6	43.3	106.0	0.0078	0.1484	0.4469	1.0000	0.0391	0.2143
Betaine	70.6	55.0	113.0	246.5	41.5	432.8	14.2	13.3	376.9	0.0156	0.2041	0.6721	1.0000	0.0078	0.0983
Carnitine	53.0	7.6	181.8	228.3	18.7	308.0	51.1	11.2	6859.7	0.0156	0.2041	0.5541	1.0000	0.2334	0.5741
Choline	75.3	19.8	1582.9	681.8	104.7	9623.0	96.3	26.4	344.6	0.0078	0.1484	0.4609	1.0000	0.0078	0.0983
Citrulline	143.0	45.6	752.2	975.9	123.0	1763.5	262.8	86.8	1127.1	0.0234	0.2333	0.0547	1.0000	0.0781	0.3154
Cyclohexylamine	1.9	0.9	3.8	0.9	0.8	1.3	1.9	1.0	4.0	0.0346	0.2634	0.7998	1.0000	0.0515	0.2528
Gln	547.4	255.3	2555.8	1679.4	554.3	5837.7	404.7	147.5	2187.5	0.0078	0.1484	0.1484	1.0000	0.0078	0.0983
Glucuronate	692.0	692.0	692.0	645.8	108.8	2866.0	449.2	57.3	841.1	0.0078	0.1484	0.3711	1.0000	0.0078	0.0983
Glutarate	2199.2	648.2	3054.6	519.2	102.7	2843.4	1451.8	408.9	4712.8	0.0391	0.2634	0.9453	1.0000	0.0156	0.1393
Gly	1109.2	370.7	39719.8	12856.5	1106.7	64448.3	1071.3	219.1	50755.4	0.0156	0.2041	0.8438	1.0000	0.0156	0.1393
Guanidinoacetate	81.1	7.7	93.3	76.2	53.5	2347.3	53.1	10.1	88.8	0.0391	0.2634	0.7874	1.0000	0.0360	0.2142
Ile	142.2	61.6	7647.9	6195.0	111.8	12163.1	131.4	64.9	8093.3	0.0078	0.1484	1.0000	1.0000	0.0078	0.0983
Imidazole-4-acetate	29.5	9.7	50.9	21.1	11.1	31.0	34.1	9.5	508.2	0.0360	0.2634	0.4469	1.0000	0.0225	0.1730
Leu	180.3	93.6	14887.6	13398.8	163.9	25732.8	209.1	125.2	17715.5	0.0156	0.2041	0.7422	1.0000	0.0078	0.0983
Lys	5216.9	1775.5	8243.1	11865.9	4083.9	51813.6	5674.0	1418.0	8843.3	0.0234	0.2333	1.0000	1.0000	0.1484	0.4125
Malate	142.3	34.8	461.1	750.5	158.8	2190.6	137.6	57.6	320.7	0.0078	0.1484	0.8885	1.0000	0.0078	0.0983
Met	94.2	41.4	2503.8	884.8	47.2	5156.0	84.1	49.1	3570.7	0.0391	0.2634	0.9453	1.0000	0.0391	0.2143
Methionine sulfoxide	73.0	25.6	314.7	259.8	54.6	534.5	75.4	24.2	549.3	0.0494	0.3226	0.5469	1.0000	0.4609	0.7417
N-Methylalanine	14.8	10.9	26.2	12.9	6.4	14.5	21.9	6.6	30.6	0.0140	0.2041	0.3125	1.0000	0.0078	0.0983
o-Acetylcarnitine	20.5	13.0	35.0	61.3	16.2	323.6	2538.7	2538.7	2538.7	0.0360	0.2634	1.0000	1.0000	0.4017	0.7104
Ornithine	258.6	63.9	1927.6	2239.6	261.3	5614.7	194.9	45.4	2067.2	0.0078	0.1484	0.5469	1.0000	0.0234	0.1730
Phe	113.1	38.3	11390.1	6958.7	105.1	15807.2	100.4	65.0	14544.1	0.0225	0.2333	0.6740	1.0000	0.0547	0.2528
Pimelate	210.5	32.8	421.1	207.5	184.2	262.1	151.2	35.0	441.3	0.0234	0.2333	0.8438	1.0000	0.0156	0.1393
Pipecolate	399.4	63.5	877.5	44.2	15.7	181.4	477.9	60.4	1064.6	0.0078	0.1484	0.7422	1.0000	0.0078	0.0983
Succinate	1756.9	966.1	46418.6	50951.9	1014.5	96369.9	1562.0	438.7	79882.2	0.0391	0.2634	0.9453	1.0000	0.0391	0.2143
Trp	113.6	51.5	3164.5	688.0	65.7	3358.1	91.8	37.1	7998.4	0.0234	0.2333	0.9453	1.0000	0.3125	0.6621
Tyr	560.4	201.5	15741.2	5126.9	329.6	16119.8	445.9	157.2	18964.3	0.0391	0.2634	0.9453	1.0000	0.0781	0.3154
Val	422.7	203.3	20646.0	13500.5	336.1	24902.8	452.7	212.4	23332.0	0.0078	0.1484	0.9453	1.0000	0.0078	0.0983
2-Hydroxybutyrate	487.4	304.9	669.9	707.4	185.7	1483.0	352.3	85.7	619.0	0.0360	0.2634	0.7893	1.0000	0.0346	0.2142
3-Hydroxybutyrate	259.8	152.4	735.7	635.9	252.4	1633.6	185.9	101.7	693.9	0.0078	0.1484	0.1056	1.0000	0.0078	0.0983
5-Aminolevulinate	15.5	9.6	23.5	12.5	9.9	17.5	15.5	8.2	24.4	0.0346	0.2634	0.2476	1.0000	0.2041	0.5138

Note. mol per 1 g of human feces (nmol/g), SD, standard deviation, NA, not applicable.

## Discussion

In the present study, we evaluated the effects of bowel prep on the stability of the gut microbiome and metabolome as well as its impact on recovery. We confirmed previous results showing that the gut microbiome is affected transiently by bowel prep but recovers quickly within 14 days. For the gut metabolome profile, we firstly demonstrated that 32 metabolites were significantly changed immediately after bowel prep but recovered after 14 days. Our findings suggest that a high-volume lavage solution is a transient and not major disrupter of the colonic ecosystem. In microbiome and metabolome studies, fecal sampling should be avoided immediately after bowel prep.

Our findings are consistent with the immediate effect of bowel prep on the gut microbiota shown in previous studies^6,7,9^. Jalanka *et al*.^9^ examined gut microbial composition using microarrays in 23 healthy volunteers before and immediately after bowel prep and at follow-up 14 and 28 days after the prep. The composition of the microbiota was affected immediately after lavage, but the unique composition and total bacterial load returned to baseline levels after 14 days. Sonnenburg *et al*.^11^ recently reported that bowel prep for 6 days triggered an immune response, some changes in gut bacteria, and short-term changes in the gut cells in mice, but 14 days after stopping the bowel prep, there was less bacterial diversity than before the course of bowel prep^11^. Their results and our findings suggest that bowel prep does affect the gut microbiome, but that the overall composition recovers to baseline within 14 days.

Nishimoto *et al*.^8^ evaluated gut microbiome composition in 8 healthy Japanese subjects before and after bowel prep with laxatives, the same way as in our study, but showed a relatively high correlation efficient at 0.88, which is higher than ours, between fecal samples before and immediately after bowel prep. The reason for this discrepancy is unclear, but the study design and statistical method might affect the results. For example, 2 of their 8 subjects were excluded from analysis due to insufficient DNA in the first fecal samples immediately after bowel prep. The lack of sufficient DNA may be attributable to the bowel prep given that an earlier study indicated the quantity of microbial DNA in feces collected immediately after prep was on average 34.7-fold lower than in the feces at baseline (p < 0.01)^9^. Although Nishimoto *et al*.’s study and ours have common findings in that there were no significant differences in the taxonomic abundance of 20 dominant genera before and after bowel prep^8^, they did not perform an in-depth analysis focused at the OTU level.

For appropriate sample collection in microbiome and metabolome studies, immediate freezing on dry ice or storage at −80 °C is standard procedure^12^. However, this can be challenging in clinical practice. Therefore, some researchers use post-bowel prep fecal samples for microbiome studies because subjects undergoing oral bowel prep in hospital invariably provide fecal samples and samples can be preserved at −80 °C immediately after collection on the colonoscopy day. Nevertheless, taking our finding of an immediate effect of bowel prep on the microbiota together with the previous finding of a long-lasting (1-month) effect^5^ suggests that fecal sampling after bowel prep is best avoided.

The strengths of the present study are that we could evaluate the gut microbiota in 70 fecal samples at different collection times from no-procedure controls as well as the bowel prep group. We were also able to measure 220 metabolites in fecal samples using CE-TOF-MS. To our knowledge, this is the first study to show the effect of bowel prep on the gut metabolome. However, there are several limitations of this study. First, in terms of the generalizability of our results to other populations, differences between subjects, physique, and countries may need to be considered. For example, the effects of bowel prep on the gut microbiota in patients with inflammatory bowel disease might be different from those in healthy subjects^4^. Second, in microbiome analysis, there were some differences in the collection methods between the control and bowel prep groups, such as different study period, sample transport, freezing, and preservation. However, a PCoA scatter plot showed that it was not divided into separate clusters between the two groups, suggesting that these differences in collection had a negligible impact on the microbiome analysis. Third, we did not have any controls in the metabolic analysis, and we could show only the metabolic changes before and after bowel prep. However, we found transient changes in the gut metabolome by bowel prep, although these changes recovered shortly after the preparation. Fourth, 3,000 reads per sample might have been too low to analyze the fecal samples, but Good’s coverage index was high in the control and bowel prep groups, suggesting that the microbiome analysis was sufficient to meet our objectives.

In conclusion, bowel preparation has a profound effect on the gut microbiome and metabolome, but the overall composition recovers to baseline within 14 days of bowel prep. Bowel cleansing before colonoscopy appears to be a safe procedure from the viewpoint of the human gut microbial ecosystem. To properly conduct studies of the human gut microbiome and metabolome, fecal sampling immediately after bowel prep should be avoided.

## Methods

### Study design, setting, and participants

Eight subjects (5 men; mean age 32.8 years; mean body mass index (BMI) 21.1) who were scheduled to undergo bowel prep were recruited from the National Center for Global Health and Medicine (NCGM), Tokyo, Japan. Data with DNA samples of 23 control subjects who did not undergo the procedure (5 men; mean age 22.0 years; mean BMI 20.2) were obtained from a published paper^13^. Baseline characteristics were shown in Supplementary Table 4. None of the subjects had any underlying disease or were treated with antibiotics, immunosuppressants, or antacids during the preceding 3 months. This study was approved by the Ethics Committee of the National Center for Global Health and Medicine, Japan (approval No 2014) and was implemented in accordance with the provisions of the Declaration of Helsinki. Written informed consent was obtained from all the participants invlolved in this study.

### Sample collection in the bowel prep and control groups

**Figure 5:**
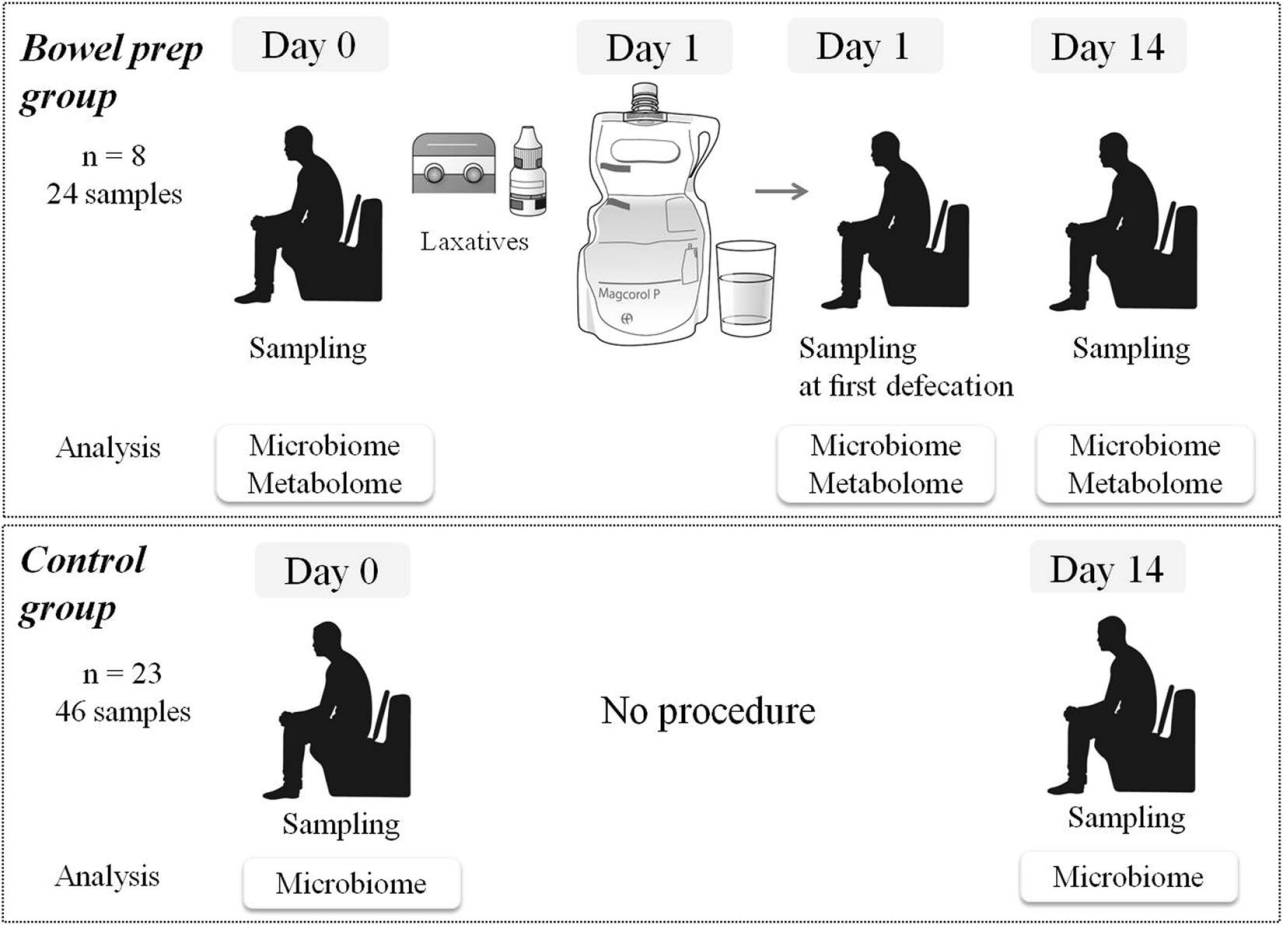
Timing of fecal sample collection in the bowel prep group and control group.

In the bowel prep group, we collected 24 fecal samples before, during, and after bowel prep (Fig. 5). All samples in bowel prep group were collected at our hospital using polypropylene tubes and preserved at −80 °C within 15 min of defecation until the time of analysis. Briefly, fecal samples were collected before laxatives were administered and bowel preparation (Day 0). Subjects then ate a commercial low-residue meal and took laxatives that included 0.67–1.0 mL sodium picosulfate hydrate (Laxoberon; Teijin Pharma Co., Ltd., Tokyo, Japan) and 12 mg sennoside (Sennoside; Sawai Pharma Co., Ltd., Osaka, Japan) at night one day before bowel preparation (Day 0). On the morning of the next day (Day 1), subjects underwent bowel prep with a 2-L high-volume lavage solution (Magcorol P; Horii Pharma Industries, Ltd., Osaka, Japan, containing magnesium citrate 68 g)^14^, to be drunk within 4 h. Fecal samples were collected immediately at the first defecation following oral administration of the high-volume lavage (Day 1). Colonoscopies were scheduled to be performed after 13:00. Fecal samples were then collected 14 days after the bowel prep.

In the control group, 46 fecal samples were collected at Day 0 and Day 14 (Fig. 5). Freshly collected feces were transported to the laboratory under anaerobic conditions in an AnaeroPack (Mitsubishi Gas Chemical Company, Inc., Tokyo, Japan) at 4 °C, were immediately frozen in liquid nitrogen in phosphate-buffered saline containing 20% glycerol, and stored at −80 °C until analysis^13^.

In both groups, the subjects were specifically asked not to alter their diet, and none of the subjects took any antibiotics, antacids, or probiotics during the study period.

### Bacterial DNA extraction and 16S rRNA gene amplicon sequencing

To extract fecal bacterial DNA, we used an enzymatic lysis method with lysozyme (Sigma-Aldrich Co., St. Louis, MO) and achromopeptidase (Wako Pure Chemical Industries, Ltd, Osaka, Japan) for all subjects, as described previously^13^. To amplify the bacterial 16S rRNA gene V3–V4 regions, we used the 16S amplicon PCR forward primer (5′-TCGTCGGCAGCGTCAGATGTGTATAAGAGACAGCCTACGGGNGGCWGCAG-3′) and the 16S amplicon PCR reverse primer (5′-GTCTCGTGGGCTCGGAGATGTGTATAAGAGACAGGACTACHVGGGTATCTAATCC-3′), with adaptor sequences for Illumina indexing. PCRs were run for 25 cycles, using the KAPA HiFi HotStart ReadyMix PCR Kit (Nippon Genetics Co., Ltd, Tokyo, Japan). Amplicons were purified with AMPure^®^ XP magnetic purification beads (Beckman Coulter, Inc., Brea, CA) and quantified with 4200 TapeStation (Agilent Technologies Japan, Ltd., Tokyo, Japan). Equal amounts of amplicons from all the samples were sequenced with the MiSeq System (Illumina, Inc., Tokyo Japan), according to the manufacturer’s instructions^13^. In the control group, we re-analyzed the 16S rRNA sequence at the V3-V4 region in the same way as for the bowel prep group from preserved DNA samples and did not use any previously obtained data^13^.

### Metabolome analysis

We performed capillary electrophoresis time-of-flight mass spectrometry (CE-TOF-MS) as described previously^15^. Fecal samples were lyophilized using a VD-800R lyophilizer (TAITEC, Saitama, Japan) for 24 h. Freeze-dried feces were disrupted with 3.0-mm Zirconia Beads (Bio Medical Science, Tokyo, Japan) by vigorous shaking (1500 rpm for 10 min) using a Shake Master (Bio Medical Science). Fecal metabolites were extracted using the methanol:chloroform: water extraction protocol. CE-TOF-MS experiments were performed using a CE System, a G3250AA LC/MSD TOF System, a 1,100 Series Binary HPLC Pump, a G1603A CE-MS adapter, and a G1607A CE-ESI-MS Sprayer Kit (all Agilent Technologies, Santa Clara, CA). We did not have any controls in the metabolic analysis, and we analyzed only the metabolic changes before and after bowel prep.

### Data analysis

All data analysis was performed in the same manner as described for an established pipeline^13,16^. After the quality of the filter-passed reads with average quality values exceeding 25 was checked for chimeras, the taxonomy of the high-quality reads was assigned using three public databases: the Ribosomal Database Project version 10.27, CORE (http://microbiome.osu.edu/), and a reference genome sequence database obtained from the National Center for Biotechnology Information FTP site (ftp://ftp.ncbi.nih.gov/genbank/, December 2011). We then selected those reads with BLAST matches exceeding 90% with a representative sequence in one of the three databases. From the filter-passed reads, 3,000 high-quality reads per sample were randomly chosen to minimize overestimation of species richness in the clustering due to intrinsic sequencing error, as previously reported^13^. Good’s coverage index accounting for 95.2% indicated that 3,000 reads were sufficient to evaluate the overall species richness and diversity. Mean (standard deviation) of Good’s coverage index was high in each group: 94.9 (1.70) in Day0 control samples, 95.7 (1.18) in Day14 control samples, 94.2 (1.84) in Day0 bowel prep samples, 94.6 (1.62) in Day1 bowel prep samples, and 93.6 (2.03) in Day14 bowel prep samples. After both primer sequences were removed, the reads were sorted and grouped into operational taxonomic units (OTUs) with a sequence identity threshold of 97%. The taxonomic assignment of each OTU was made with the GLSEARCH program. Taxonomic groups with relative abundances exceeding 0.1% in any subject were included in subsequent analyses. From the 70 fecal samples, a total of 898,035 high-quality reads were obtained, ranging from 9,152 to 9,840 reads per sample. All 3,000 filter-passed reads of the 16S V3–V4 sequences analyzed in this study were deposited in the DDBJ/GenBank/EMBL database under accession numbers DRA 007110.

The α-diversity of microbial communities in each sample were evaluated using the Shannon diversity index. Spearman’s correlation coefficients were used to compare the overall bacterial and metabolomic compositions between the different sample collection times. For the UniFrac distance analysis, phylogenetic tree-based metrics were used to measure the differences in overall bacterial composition at the different sample collection times^17^. A Wilcoxon’s signed-rank test was used to evaluate within-subject differences in bacterial and metabolic composition across the different sample collection times (e.g. UniFrac distance or coefficient between Day 0 and Day 1 samples vs between Day 0 and Day 14 samples). A Wilcoxon rank sum test was used to assess the difference in bacterial composition between group differences within subjects (e.g. UniFrac distance or coefficient between the bowel prep group vs controls). PCoA was applied for the analysis of the microbial and metabolic data. We used unit variance scaling as the data description measure for metabolites^18^, with the standard deviation as the scaling factor^18^. Values of p < 0.05 were considered statistically significant. All statistical analyses were performed with the R software package (v3.2.2).

## Supplementary information

Supplementary Figures

Supplementary Tables

## Data Availability

All 3,000 filter-passed reads of the 16S rRNA sequences analyzed in this study were deposited in the DDBJ/GenBank/EMBL database under accession numbers DRA 007110.
