# Supplementary material for: Effects of bowel preparation on the human gut microbiome and metabolome
Source: Sci Rep. 2019 Mar 11;9:4042. doi: 10.1038/s41598-019-40182-9 (PMC6411954; doi:10.1038/s41598-019-40182-9)
Supplement: Supplementary file 1 — Supplementary Figures [file 41598_2019_40182_MOESM1_ESM.docx]

**Title: Effects of bowel preparation on the human gut microbiome and metabolome**

Naoyoshi Nagata, MD, PhD^1^, Mari Tohya, PhD^2,3^, Shinji Fukuda, PhD^4-7^, Wataru Suda, PhD^11^, Suguru Nishijima, PhD^9,10^, Fumihiko Takeuchi, PhD^8^, Mitsuru Ohsugi, MD^12^, Tetsuro Tsujimoto, MD, PhD^12^, Tomoka Nakamura, MD^12^, Akira Shimomura, MD^1^, Naohiro Yanagisawa, MD^1^, Yuya Hisada, MD^1^, Kazuhiro Watanabe, MD^1^, Koh Imbe, MD, PhD^1^, Junichi Akiyama, MD^1^, Masashi Mizokami, MD, PhD^13^, Tohru Miyoshi-Akiyama, PhD^2^, Naomi Uemura, MD, PhD^13^, and Masahira Hattori, PhD^10,11^

^1^Department of Gastroenterology and Hepatology, National Center for Global Health and Medicine.

^2^Pathogenic Microbe Laboratory, Research Institute, National Center for Global Health and Medicine.

^3^Department of Microbiology, Juntendo University School of Medicine, Tokyo, Japan

^4^Institute for Advanced Biosciences, Keio University.

^5^Intestinal Microbiota Project, Kanagawa Institute of Industrial Science and Technology

^6^Transborder Medical Research Center, University of Tsukuba.

^7^PRESTO, Japan Science and Technology Agency

^8^Department of Gene Diagnostics and Therapeutics, National Center for Global Health and Medicine.

^9^Computational Bio-Big Data Open Innovation Lab., National Institute of Advanced Science and Technology.

^10^Graduate School of Advanced Science and Engineering, Waseda University, Tokyo, Japan

^11^RIKEN Center for Integrative Medical Sciences.

^12^Department of Diabetes, Endocrinology, and Metabolism, Center Hospital, National Center for Global Health and Medicine.

^13^Department of Gastroenterology and Hepatology, National Center for Global Health and Medicine, Kohnodai Hospital.

**Correspondence:** Naoyoshi Nagata, MD, PhD. Department of Gastroenterology and Hepatology, National Center for Global Health and Medicine, 1-21-1 Toyama, Shinjuku-ku, Tokyo 162-8655, Japan.

Tel: +81-03-3202-7181; Fax: +81-03-3207-1038

E-mail: [nnagata_ncgm@yahoo.co.jp](mailto:nnagata_ncgm@yahoo.co.jp)

**Supplementary Figure1. Relative differences in the abundances of four phyla in regular feces (Day 0), first feces immediately after prep (Day 1), and feces 14 days after bowel prep (Day 14).**

**
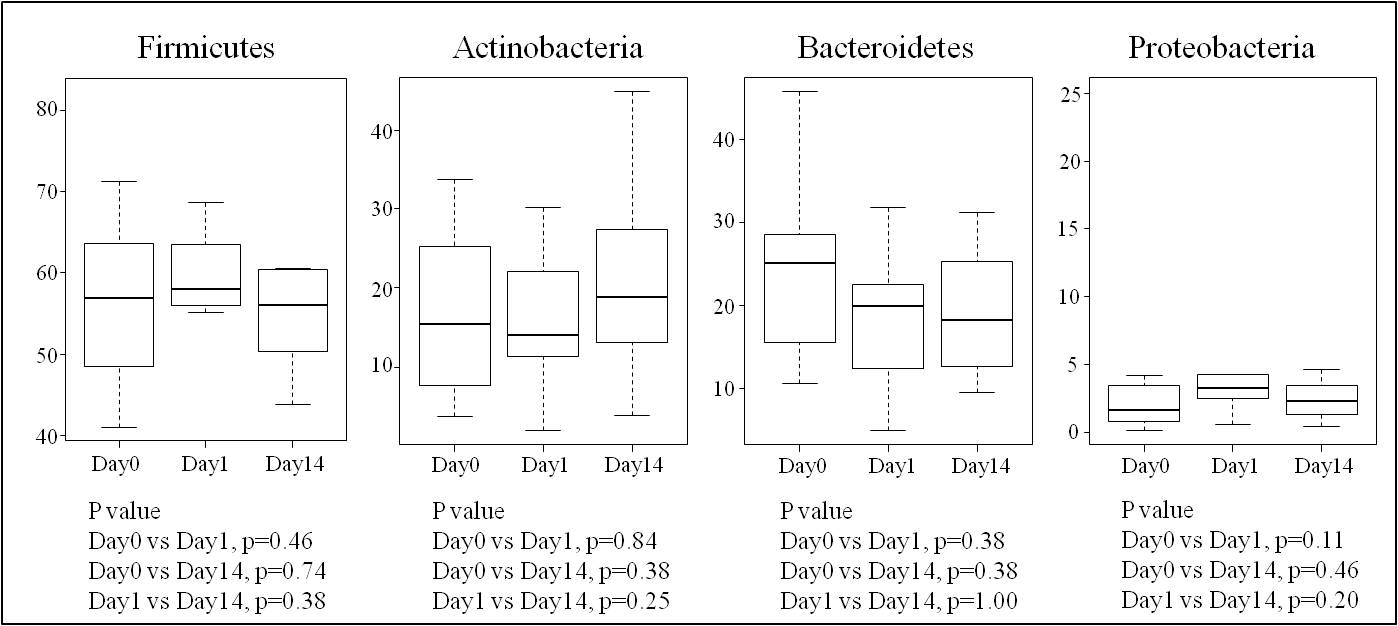
**

Note: Boxes represent the interquartile range (IQR) and lines inside show the median. Whiskers denote the lowest and highest values within 1.5 times the IQR.

**Supplementary Figure 2. Number of changed operational taxonomic units (OTUs) between Day 1/Day 0 and Day 14/Day 0 read ratios with the given thresholds.**

**
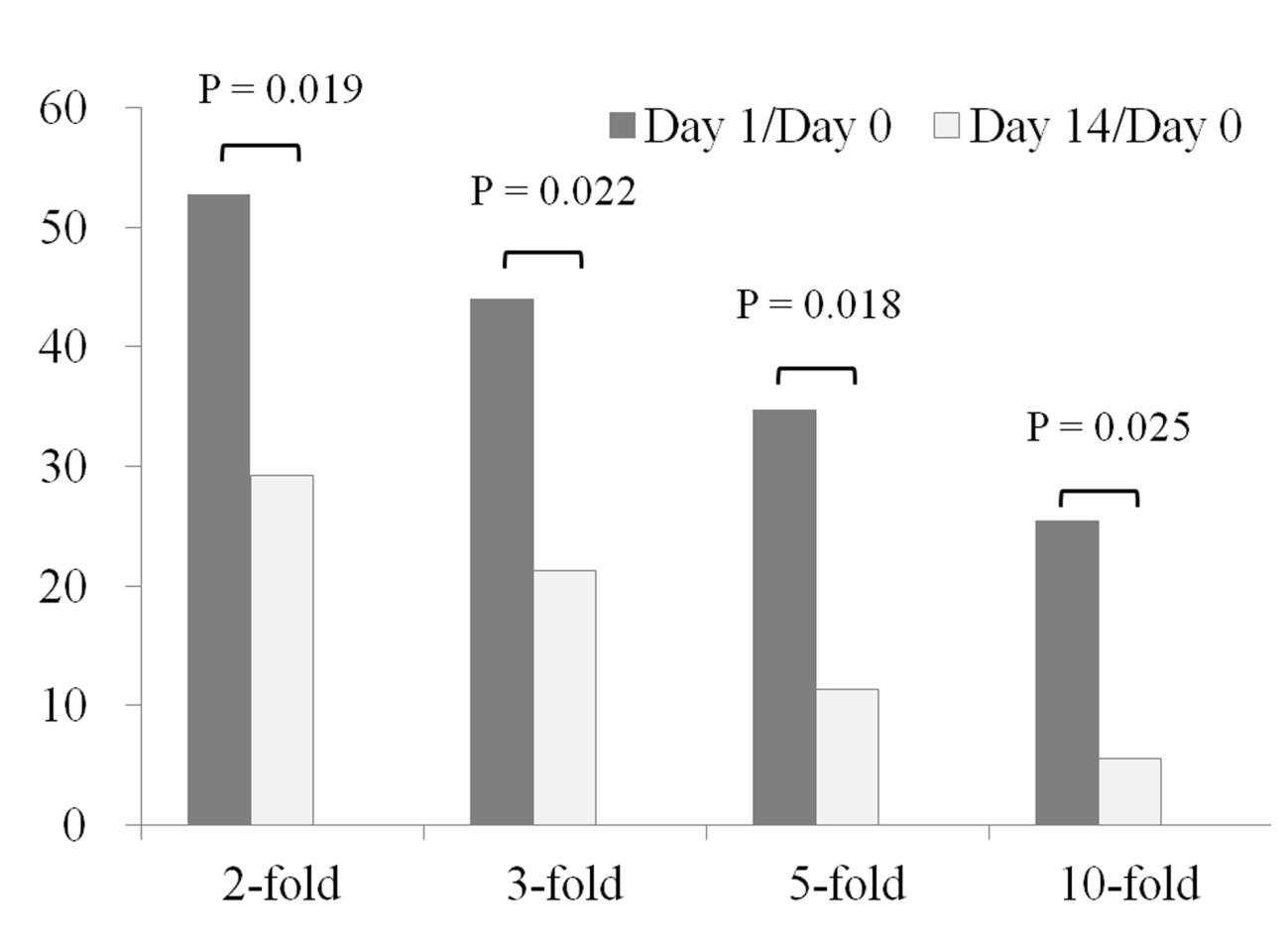
**

**Note:** Bar graph shows mean values. OTUs with ≥ 3 reads for at least one of the three samples at Day 0, Day 1, and Day 14 were analyzed. Changed OTUs were defined as OTUs with increased or decreased Day 1/Day 0 read ratios ≥2-fold, 3-fold, 5-fold, and 10-fold. Increased or decreased Day 14/Day 0 read ratios with the given thresholds were also analyzed.
